# The Effect of Perceived Social Support on Frailty and Medication Adherence in Patients With Chronic Obstructive Pulmonary Disease

**DOI:** 10.1111/jocn.70481

**Published:** 2026-07-31

**Authors:** N. Özdemir

**Affiliations:** ^1^ Depertmant of Occupational Health and Safety, Faculty of Health Sciences University of Mus Alparslan Muş Turkey

**Keywords:** chronic obstructive pulmonary disease, frailty, medication adherence, nursing, social support

## Abstract

**Background:**

Chronic obstructive pulmonary disease is a progressive respiratory condition associated with significant morbidity and long‐term treatment requirements. Disease management is influenced not only by clinical factors but also by psychosocial determinants such as perceived social support, frailty, and medication adherence.

**Aims:**

This study aimed to examine the relationships between perceived social support, frailty, and medication adherence in patients with chronic obstructive pulmonary disease and to determine whether perceived social support was associated with frailty and medication adherence.

**Methods:**

This cross‐sectional study was conducted with 126 patients diagnosed with chronic obstructive pulmonary disease. Data were collected using a sociodemographic and clinical characteristics form, the Multidimensional Scale of Perceived Social Support, the Medication Adherence Report Scale, and the Edmonton Frail Scale.

**Results:**

The mean perceived social support score was 48.75 ± 13.07, the mean frailty score was 8.10 ± 3.03, and the mean medication adherence score was 18.30 ± 4.20. Perceived social support was positively correlated with medication adherence (*r* = 0.285, *p* = 0.001) and negatively correlated with frailty (*r* = −0.421, *p* < 0.001). Regression analyses showed that perceived social support was significantly associated with lower frailty (*B* = −0.10, *β* = −0.42, *p* < 0.001) and positively associated with medication adherence (*B* = 0.09, *β* = 0.29, *p* < 0.001).

**Conclusion:**

Perceived social support may be an important factor associated with frailty and medication adherence in patients with chronic obstructive pulmonary disease.

**Impact:**

These findings highlight the importance of integrating psychosocial assessment into nursing care and support the consideration of family‐centered interventions in the care of patients with chronic obstructive pulmonary disease.

**Implications for Practice:**

These findings suggest that nurses and healthcare professionals should routinely assess perceived social support in patients with chronic obstructive pulmonary disease. Family involvement and psychosocially oriented nursing approaches may be considered as part of comprehensive patient care. However, the effectiveness of these approaches should be confirmed in future longitudinal and interventional studies.

**No Patient or Public Contribution:**

Patients participated in the study only as respondents. They were not involved in the design, conduct, analysis, interpretation or dissemination of the research.

## Key Points

- Perceived social support was associated with lower frailty in patients with COPD.
- Higher perceived social support was associated with better medication adherence.
- Routine assessment of perceived social support may help identify patients requiring additional support.
- The findings support considering family‐centred psychosocial care as part of comprehensive COPD nursing practice.

## 1 Introduction

Chronic Obstructive Pulmonary Disease (COPD) is a chronic respiratory condition characterized by progressive airflow limitation and associated with high morbidity and mortality. The disease course leads to symptoms such as dyspnea, fatigue, and reduced physical capacity, which restrict individuals' activities of daily living and require long‐term treatment (Özdemir and Enç 2022). Effective disease management and treatment adherence depend not only on medical interventions but also on psychosocial factors. Among these, perceived social support—defined as the emotional, informational, and instrumental assistance received from family, friends, and close social networks—is recognized as a key determinant in chronic disease management (Vahedparast et al. 2018).

In the literature, social support has been shown to improve self‐care behaviours, enhance treatment adherence, and improve quality of life in chronic diseases (Chen et al. 2017; Bhattarai et al. 2020). However, findings specific to patients with COPD are limited and are mostly focused on particular sources of social support (e.g., family support). Moreover, a large proportion of existing studies conceptualize social support as a unidimensional construct; thus, there is a paucity of research comprehensively examining the effects of different components of perceived social support (family, friends, and significant others) on disease‐related outcomes (Vahedparast et al. 2018; Liu et al. 2024; Johansson et al. 2025). This gap highlights the need to more thoroughly examine the associations between the multidimensional structure of social support and disease management in patients with COPD. Accordingly, adopting a biopsychosocial model rather than a purely biomedical approach is essential in COPD management to better understand individuals' health status and to promote holistic improvement in care outcomes (Sundar 2024).

In recent years, frailty—an important concept in chronic disease management—has been defined as a clinical condition characterized by a decline in physiological reserve and reduced resistance to stressors (Sieber 2017). In patients with COPD, frailty is known to have a high prevalence and is strongly associated with disease severity, hospitalization, and mortality (Wang et al. 2023). Moreover, the increased physical and cognitive limitations observed in frail individuals may negatively affect medication adherence by complicating daily medication management (Bonikowska et al. 2022). It has been suggested that frailty may serve as a more practical and equally powerful indicator of medication adherence in older patients compared to comorbidity (Lapi et al. 2025). However, the direct impact of frailty on medication adherence in patients with COPD has not yet been clearly established, representing an important area of research for improving disease outcomes (Wang et al. 2023).

Medication adherence is one of the most critical determinants of treatment success in COPD management, and its clinical importance is further emphasized by evidence showing that regular inhaler use plays a key role in symptom control, reduction of exacerbation frequency, and prevention of hospitalizations (Vauterin et al. 2024). However, medication adherence in patients with COPD is frequently suboptimal due to factors such as polypharmacy, difficulties in inhaler technique, lack of disease‐related knowledge, and psychosocial determinants (O'Toole et al. 2022; Vauterin et al. 2024), and this has been associated with increased risks of hospitalization, exacerbations, and mortality (Bhattarai et al. 2020; Vestbo et al. 2009). Although the literature suggests that reduced social support in older adults may negatively affect medication adherence (Costello et al. 2016), studies specifically examining the relationship between perceived social support and medication adherence in patients with COPD remain highly limited.

The present study aimed to examine perceived social support through its subdimensions (family, friends, and significant others) and to investigate the relationships between perceived social support, frailty, and medication adherence in patients with COPD. Specifically, the study sought to determine the levels of perceived social support, frailty, and medication adherence, to examine the relationships among these variables, and to examine the associations of perceived social support with frailty and medication adherence. By addressing these aims, this study fills an important gap in the literature regarding the multidimensional associations between perceived social support and COPD‐related outcomes. The conceptual framework of the study is presented in Figure 1.

**FIGURE 1:**
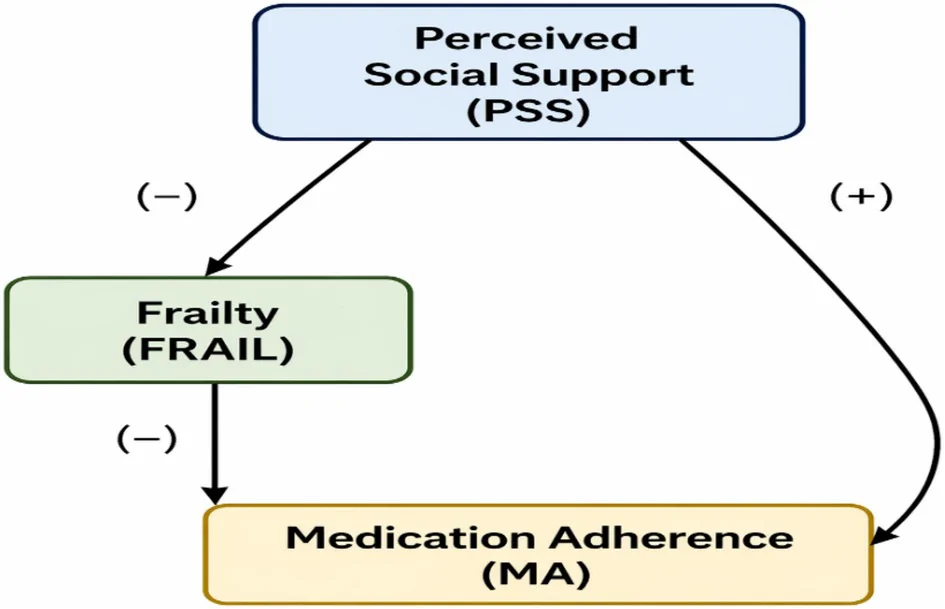
Conceptual framework of the study. [Colour figure can be viewed at wileyonlinelibrary.com]

## 2 Materials and Methods

### 2.1 Participants

This cross‐sectional, single‐center study included patients diagnosed with COPD who attended the Chest Diseases Service of Muş State Hospital during the data collection period. Participants were recruited using a convenience sampling method. Patients who met the eligibility criteria and voluntarily agreed to participate in the study were included. The study population consisted of patients who attended the outpatient clinic during the study period.

The sample size was calculated using G*Power 3.1.9.2 software. Based on previous literature, a small‐to‐moderate correlation (*r* = 0.22) between perceived social support and related variables in patients with COPD was assumed (Chen et al. 2017). With a significance level of 0.05 (*α* = 0.05) and a statistical power of 80% (1–*β* = 0.80), the minimum required sample size was determined to be 120 participants. The study was completed with 126 patients, and all analyses were conducted on this sample. In addition, the sample size was considered adequate for the linear regression analyses because only one independent variable (perceived social support) was included in each regression model, and the final sample exceeded the minimum sample size recommended for regression analysis (Green 1991). The STROBE guidelines were utilized in reporting this study. The STROBE reporting checklist is provided as Data S1.

#### 2.1.1 Sample Selection Criteria

Inclusion criteria were as follows: being 18 years of age or older, having a confirmed diagnosis of COPD, being in a clinically stable condition (not in an acute exacerbation period), being able to communicate effectively, and voluntarily agreeing to participate in the study.

Exclusion criteria included being in an acute exacerbation phase, receiving intensive care treatment, having severe cognitive or psychiatric disorders that could interfere with data collection, and incomplete completion of the study questionnaires.

### 2.2 Research Location and Characteristics

The study was conducted in the Chest Diseases Service, Muş State Hospital, Muş, Türkiye. Data collection was carried out between July 2024 and August 2025.

### 2.3 Data Collection and Questionnaires

The study's independent variable was perceived social support. The dependent variables were frailty and medication adherence. In addition, sociodemographic and clinical characteristics of the participants (e.g., age, gender, marital status, education level, income status, smoking status, comorbidities, and number of medications used) were considered as descriptive and potential confounding variables.

#### 2.3.1 Demographic and Clinical Characteristics of the Patients

This form, developed by the researcher in accordance with the relevant literature, consists of a total of 13 items designed to assess the sociodemographic and clinical characteristics of the participants. The form includes questions regarding age, gender, marital status, living arrangements, educational and employment status, income level, smoking status, presence of comorbidities, history of hospitalization, medication use, and the number of medications used (O'Toole et al. 2022; Chen et al. 2017; Özdemir and Enç 2022).

#### 2.3.2 Multidimensional Scale of Perceived Social Support (MSPSS)

The Multidimensional Scale of Perceived Social Support, developed by Zimet et al. (1988), was used to assess the level of perceived social support. The Turkish validity and reliability of the scale were established by Eker and Arkar (1995). The scale consists of 12 items rated on a 7‐point Likert scale and includes three subdimensions: family, friends, and significant other support. The total score ranges from 12 to 84, with higher scores indicating higher levels of perceived social support. In the present study, the Cronbach's alpha coefficient was 0.90 for the Multidimensional Scale of Perceived Social Support, indicating excellent internal consistency.

#### 2.3.3 Edmonton Frail Scale (EFS)

The Edmonton Frail Scale, developed by Rolfson et al. (2006) was used to assess the level of frailty in older adults. The Turkish validity and reliability of the scale were established by Aygör (2013). The scale consists of 11 items and is scored within a range of 0 to 20. Based on the total score, frailty is classified as follows: 0–4 indicates not frail, 5–6 vulnerable, 7–8 mildly frail, 9–10 moderately frail, and ≥ 11 severely frail. In the present study, the Cronbach's alpha coefficient for the EFS was 0.73, indicating acceptable internal consistency.

#### 2.3.4 Medication Adherence Report Scale (MARS)

The Medication Adherence Report Scale, developed by Horne (2000), was used to assess medication adherence. The Turkish validity and reliability of the scale were established by (Şen et al. 2019). The scale consists of 5 items rated on a 5‐point Likert scale (5 = never, 1 = always). The total score ranges from 5 to 25, with higher scores indicating better medication adherence. In the present study, the Cronbach's alpha coefficient for the MARS was 0.88, indicating good internal consistency.

Data were collected via face‐to‐face interviews conducted by the researchers. Completing the questionnaires took approximately 10–15 min.

### 2.4 Statistical Method

#### 2.4.1 Data Analysis

Data were analysed using SPSS (Statistical Package for the Social Sciences) version 27.0. The Kolmogorov–Smirnov test was used to assess the normality of data distribution. Descriptive statistics (frequency, percentage, mean, and standard deviation) were used to summarize the data. The internal consistency of the scales was evaluated using Cronbach's alpha coefficient. Spearman's correlation analysis was used to examine the relationships between variables. Linear regression analysis was performed to identify factors affecting the dependent variables.

Simple linear regression analyses (Enter method) were performed to examine the associations between perceived social support and the dependent variables (frailty and medication adherence). No adjustment for potential confounding variables (e.g., age, sex, comorbidities, or disease stage) was performed because the aim of the regression analyses was to evaluate the independent association of perceived social support with the study outcomes. Prior to regression analyses, the assumptions of normality and multicollinearity were assessed using the Kolmogorov–Smirnov test and tolerance/variance inflation factor (VIF) values, respectively. A *p*‐value of < 0.05 was considered statistically significant.

## 3 Results

### 3.1 Demographic and Clinical Characteristics

The demographic and clinical characteristics of the patients with COPD are presented in Table 1. A total of 126 participants were included. The mean age was 62.46 ± 14.03 years, and most participants were aged ≥ 61 years (63.5%). The majority were male (56.3%) and married (68.3%). Most participants lived with their spouse and/or children (70.6%).

**TABLE 1:** Demographic and clinical characteristics of the patients (*n* = 126).

Variable	*n*	%
Age groups (years)		
30–40	14	11.1
41–50	6	4.8
51–60	26	20.6
≥ 61	80	63.5
Mean	62.46 ± 14.03	
Marital status		
Married	86	68.3
Single	40	31.7
Sex		
Male	71	56.3
Female	55	43.7
Living arrangement		
Alone	20	15.9
With parents	10	7.9
With spouse/children	89	70.6
With caregiver	4	3.2
With relatives	3	2.4
Educational level		
Illiterate	41	32.5
Primary/secondary school	52	41.3
High school	24	19.0
University graduate	9	7.1
Employment status		
Employed	11	8.7
Unemployed	69	54.8
On leave	16	12.7
Retired	30	23.8
Income level		
Good	5	4.0
Moderate	80	63.5
Poor	41	32.5
Smoking status		
Yes	33	26.2
No	40	31.7
Former smoker	53	42.1
Comorbidity		
Yes	103	81.7
No	23	18.3
Disease stage		
Stage I	27	21.4
Stage II	45	35.7
Stage III	35	27.8
Stage IV	17	13.5
Regular medication use		
Yes	91	72.2
No	35	27.8
Reason for non‐adherence		
Forgetfulness	10	7.9
No perceived benefit	5	4.1
Multiple medications	12	9.5
Side effects	8	6.3
Number of medications per day		
1–2	23	18.3
3	27	21.4
4	27	21.4
≥ 5	49	38.9

Regarding educational status, 41.3% had primary/secondary education, 32.5% were illiterate, 19.0% had completed high school, and 7.1% were university graduates. More than half were unemployed (54.8%), while 23.8% were retired.

Most participants reported a moderate income level (63.5%). In terms of smoking status, 42.1% were former smokers, 31.7% had never smoked, and 26.2% were current smokers. Comorbidities were present in 81.7% of participants. Regarding disease stage, 35.7% were classified as Stage II, followed by Stage III (27.8%), Stage I (21.4%), and Stage IV (13.5%). Regular medication use was reported by 72.2% of participants.

Among reasons for non‐adherence, the most common were multiple medication use (9.5%), forgetfulness (7.9%), side effects (6.3%), and lack of perceived benefit (4.1%). In terms of daily medication use, 38.9% of participants reported using ≥ 5 medications.

### 3.2 Descriptive Statistics of the Scales

The mean scores and standard deviations of perceived social support, medication adherence, and frailty are shown in Table 2. The mean total score of the Perceived Social Support Scale was 48.75 ± 13.07 (range: 17–84). Subscale scores were 18.50 ± 4.96 for family support (range: 5–28), 15.45 ± 5.31 for friend support (range: 4–28), and 14.76 ± 5.68 for significant other support (range: 4–28). The mean Medication Adherence Scale score was 18.30 ± 4.20 (range: 4–25). The mean total score of the Edmonton Frailty Scale was 8.10 ± 3.03 (range: 1–16).

**TABLE 2:** Descriptive statistics of the scales (*n* = 126).

Scale	Min–Max	Mean ± SD
Perceived social support scale (total)	17–84	48.75 ± 13.07
Family support	5–28	18.5 ± 4.96
Friend support	4–28	15.45 ± 5.31
Significant other support	4–28	14.76 ± 5.68
Medication adherence scale	4–25	18.30 ± 4.20
Edmonton frailty scale (total)	1–16	8.10 ± 3.03

### 3.3 Distribution of Frailty Levels

Distribution of COPD patients according to frailty levels is shown in Table 3. According to the Edmonton Frailty Scale classification, 27.8% of participants were classified as severely frail, 23.0% as mildly frail, 21.4% as vulnerable, 16.7% as moderately frail, and 11.1% as not frail. The distribution of frailty levels among COPD patients is shown in Figure 2.

**TABLE 3:** Distribution of frailty levels (*n* = 126).

Frailty level	*n*	%
Not frail (0–4)	14	11.1
Vulnerable (5–6)	27	21.4
Mild frailty (7–8)	29	23
Moderate frailty (9–10)	21	16.7
Severe frailty (≥ 11)	35	27.8

**FIGURE 2:**
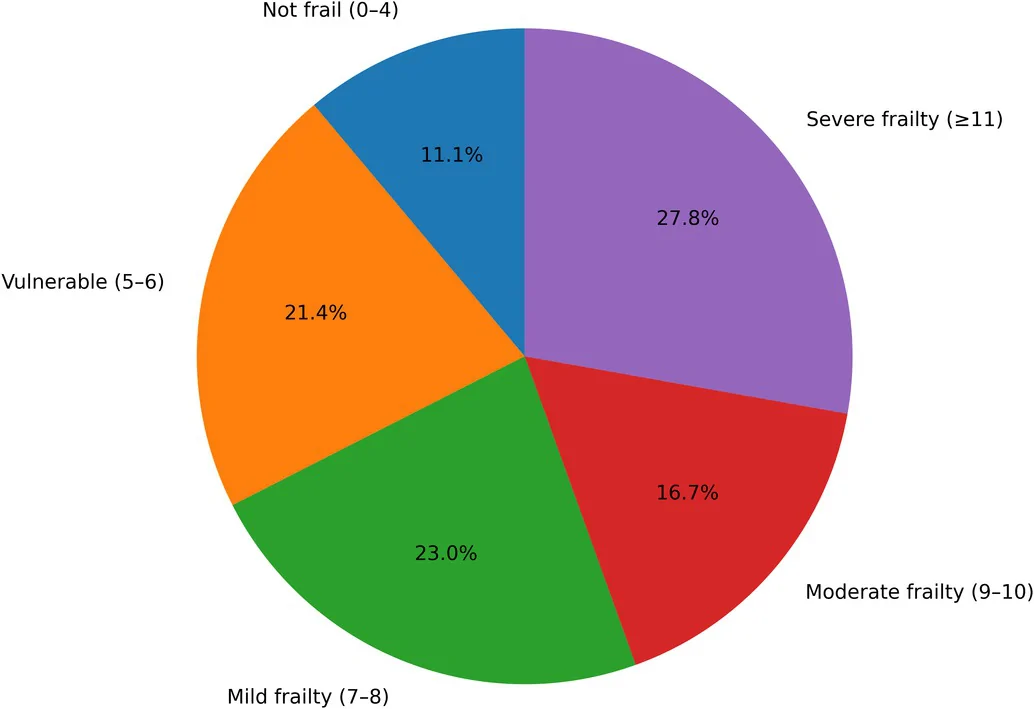
Distribution of frailty levels among COPD patients. [Colour figure can be viewed at wileyonlinelibrary.com]

### 3.4 Correlations Between Perceived Social Support, Frailty, and Medication Adherence

The relationships between perceived social support, frailty, and medication adherence are shown in Table 4. Significant positive correlations were found between medication adherence and family support (*r* = 0.416, *p* < 0.001) and total social support (*r* = 0.285, *p* = 0.001). However, medication adherence was not significantly correlated with frailty (*r* = −0.096, *p* = 0.283), friend support (*r* = 0.144, *p* = 0.108), or significant other support (*r* = 0.159, *p* = 0.076).

**TABLE 4:** Correlations between perceived social support, frailty, and medication adherence (*n* = 126).

Variables	1	2	3	4	5	6
1. Medication adherence	1					
2. Family support	0.416**	1				
3. Frailty	−0.096	−0.180*	1			
4. Friend support	0.144	0.454**	−0.429**	1		
5. Significant other support	0.159	0.249**	−0.411**	0.789**	1	
6. Total social support	0.285**	0.673**	−0.421**	0.922**	0.850**	1

* *p* < 0.05.

** *p* < 0.01.

Frailty showed significant negative correlations with family support (*r* = −0.180, *p* = 0.044), friend support (*r* = −0.429, *p* < 0.001), significant other support (*r* = −0.411, *p* < 0.001), and total social support (*r* = −0.421, *p* < 0.001).

Social support subscales were strongly interrelated, with friend support showing strong positive correlations with significant other support (*r* = 0.789, *p* < 0.001) and total social support (*r* = 0.922, *p* < 0.001), while family support was also strongly associated with total social support (*r* = 0.673, *p* < 0.001).

### 3.5 Effects of Perceived Social Support on Frailty and Medication Adherence

Effects of perceived social support on frailty and medication adherence are presented in the Table 5. Linear regression analysis showed that perceived social support was significantly associated with frailty. Higher perceived social support was associated with lower frailty levels (*β* = −0.42, *t* = −5.16, *p* < 0.001). The model indicated a significant negative effect (*B* = −0.10, SE = 0.02).

**TABLE 5:** Effects of perceived social support on frailty and medication adherence (*n* = 126).

Model	Dependent variable	Predictor	B	SE	*β*	*t*	*p*
Model 1	Frailty (mean)	Constant	12.90	0.96		13.40	< 0.001
		Perceived social support (mean)	−0.10	0.02	−0.42	−5.16	< 0.001
Model 2	Medication adherence (mean)	Constant	13.83	1.40		9.89	< 0.001
		Perceived social support (mean)	0.09	0.03	0.29	3.32	< 0.001

In Model 2, perceived social support was significantly associated with medication adherence. Higher perceived social support was associated with higher medication adherence (*β* = 0.29, *t* = 3.32, *p* < 0.001), with a positive regression effect (*B* = 0.09, SE = 0.03).

## 4 Discussion

In this study, the relationships between perceived social support, frailty, and medication adherence in patients with COPD were examined, and the association of perceived social support on these variables was evaluated. The findings suggest that psychosocial factors, beyond medical treatment, may be important in the management of COPD.

When the descriptive findings were examined, it was observed that the perceived social support levels of the participants were at a moderate level, and among the subdimensions, the highest mean score belonged to family support. This finding is consistent with the literature indicating that the caregiving process in individuals with chronic diseases is largely undertaken by family members (Shirvani et al. 2020; Cruz et al. 2017). The fact that medication adherence scores were above the average indicates a relatively acceptable level of adherence in the sample group. This finding is similar to some studies and is partially consistent with the literature reporting that medication adherence in patients with COPD is at a moderate level (Saja et al. 2025; Moradkhani et al. 2021). However, there are also studies in the literature indicating that medication adherence in patients with COPD is at a low level (O'Toole et al. 2022; Vauterin et al. 2024). These differences reveal that the results regarding medication adherence may vary across studies. It is considered that these differences may be due to variables such as the sociodemographic characteristics of the sample, level of access to healthcare services, structure of social support, and disease management characteristics (O'Toole et al. 2022; Wang et al. 2025).

In the present study, it was determined that frailty levels were high and that a large proportion of participants were in the frail or at‐risk group in terms of frailty. In particular, the high proportion of severely frail individuals supports that frailty is an important clinical problem in patients with COPD. This finding is consistent with similar studies in the literature (Wang et al. 2023; Hanlon et al. 2023) and demonstrates that frailty is a prevalent and clinically significant problem in patients with COPD.

One of the most striking findings of the study is the significant negative relationship identified between perceived social support and frailty. Both correlation and regression analyses indicated that as social support increases, frailty levels decrease. This finding suggests that higher levels of perceived social support are associated with lower levels of frailty. One possible explanation is that social support may facilitate participation in physical activity, strengthen psychological resilience, and improve access to healthcare services, all of which have been associated with lower frailty in previous studies (Jin et al. 2022). In addition, the fact that family, friend, and significant other support were all negatively associated with frailty indicates that not only family‐based support but also broader social networks play an important role in maintaining functional capacity (Lenferink et al. 2018).

Another important finding of the study is that perceived social support was positively associated with medication adherence. It was determined that as the level of social support increases, medication adherence also increases. When the subdimensions were examined, family support was found to be more strongly associated with medication adherence. This finding suggests that family members may facilitate treatment adherence through roles such as medication reminders, encouraging treatment, and actively participating in care processes (Trivedi et al. 2012). In contrast, the lack of a significant relationship between friend and significant other support and medication adherence indicates that not all components of social support are equally effective. There are also studies in the literature reporting no significant relationship between social support and medication adherence (Chen et al. 2017). These inconsistent findings suggest that the effect of social support on medication adherence may vary depending on context and the source of support. In this regard, strengthening family support may represent a promising focus for future intervention studies.

In the present study, no statistically significant relationship was found between frailty and medication adherence; however, a negative trend was observed. These findings are not fully consistent with previous studies reporting that frailty negatively affects medication adherence in older adults (Li et al. 2018). However, it is noteworthy that most of these studies were conducted in populations with chronic diseases other than COPD (Chudiak et al. 2017; Uchmanowicz et al. 2019; Li et al. 2018), limiting direct comparison with the present findings. The absence of a significant association may partly be explained by the characteristics of the study sample. Participants reported relatively high levels of perceived social support across all MSPSS subdimensions, and most were regular medication users. Furthermore, the majority lived with their spouses, children, or other family members (84.1%), suggesting that family support may have compensated for functional limitations associated with frailty by facilitating medication management. In addition, participants were recruited from a chest diseases outpatient clinic and received regular outpatient follow‐up care, which may have provided continuous professional support for treatment adherence. Together, family involvement and regular healthcare follow‐up may have attenuated the potential negative impact of frailty on medication adherence. Another possible explanation relates to the multidimensional nature of frailty and the characteristics of the assessment instrument used. The Edmonton Frail Scale is a validated multidimensional screening tool that evaluates several domains of frailty, including cognition, general health status, functional independence, social support, medication use, nutrition, mood, continence, and functional performance. While these domains reflect the clinical manifestations of frailty, the scale does not directly assess the biological and neurological mechanisms underlying the syndrome. Recent neuroimaging evidence has demonstrated that frailty in patients with COPD is associated with reduced cortical thickness in brain regions involved in motor control and cognitive processing, suggesting that frailty reflects not only physical decline but also structural brain alterations (Fukatsu‐Chikumoto et al. 2024). These findings indicate that the relationship between frailty and medication adherence may be indirect and influenced by complex interactions among cognitive function, self‐management capacity, caregiver support, and healthcare access, rather than by physical frailty alone. Taken together, these findings suggest that medication adherence in COPD is a multidimensional behaviour that cannot be explained solely by frailty but is shaped by biological, psychological, social, and healthcare‐related factors (Krousel‐Wood et al. 2021). Because relatively few studies have directly examined the relationship between frailty and medication adherence in patients with COPD, further longitudinal studies using multidimensional frailty assessments together with objective measures of medication adherence are warranted to clarify these complex relationships.

This study has several limitations. First, the cross‐sectional design does not allow for the establishment of causal relationships. In addition, participants were recruited from the chest diseases outpatient clinic of a single state hospital using a convenience sampling method, which may have introduced selection bias and limited the generalizability of the findings. Participants may differ from patients managed in primary care or those receiving home‐based care with respect to social support, functional status, and access to caregiving. Therefore, the findings should be interpreted with caution. Furthermore, all study variables were assessed using self‐reported questionnaires, which may have introduced reporting bias, including recall bias and social desirability bias. Future multicenter studies using probability‐based sampling methods are recommended to improve the external validity and generalizability of the findings.

## 5 Conclusion

In conclusion, perceived social support was significantly associated with frailty and medication adherence in patients with chronic obstructive pulmonary disease, whereas no significant association was observed between frailty and medication adherence. These findings support the importance of a biopsychosocial approach in chronic disease management. Accordingly, the assessment of social support should be considered an integral part of routine care. Nurses should systematically evaluate patients' social support systems and identify individuals with insufficient support at an early stage, directing them toward appropriate support services when appropriate. The present findings provide a rationale for future family‐centred and psychosocial interventions; however, the effectiveness of such interventions in reducing frailty and improving medication adherence should be confirmed through longitudinal and intervention studies. Future research using longitudinal and multi‐centre designs is recommended to better understand causal relationships and to validate these findings in different populations.

## Funding

The author has nothing to report.

## Ethics Statement

Ethical approval was obtained from the Scientific Research Ethics Committee of Muş Alparslan University (meeting date: 02 January 2023, decision no.: 56) and institutional permission was obtained from Muş State Hospital before the study was conducted. Written informed consent was obtained from all participants after they had been informed about the purpose and procedures of the study. The study was conducted in accordance with the ethical principles of the Declaration of Helsinki.

## Conflicts of Interest

The author declares no conflicts of interest.

## Supporting information


**Data S1:** The STROBE reporting checklist.

## Data Availability

The data that support the findings of this study are available from the corresponding author upon reasonable request.
